# SKP-SCs transplantation alleviates 6-OHDA-induced dopaminergic neuronal injury by modulating autophagy

**DOI:** 10.1038/s41419-021-03967-3

**Published:** 2021-07-05

**Authors:** Chengxiao Ma, Wen Zhang, Wengcong Wang, Jiabing Shen, Kefu Cai, Mei Liu, Maohong Cao

**Affiliations:** 1 Department of Neurology, Affiliated Hospital of Nantong University, Nantong, China; 2 Key Laboratory of Neuroregeneration of Jiangsu and Ministry of Education, Co-innovation Center of Neuroregeneration, Nantong University, Nantong, China

**Keywords:** Autophagy, Neurodegeneration, Parkinson's disease

## Abstract

Parkinson’s disease is a common neurodegenerative disease. Cell transplantation is a promising therapeutic option for improving the survival and function of dopaminergic neurons, but the mechanisms underlying the interaction between the transplanted cells and the recipient neurons remain to be studied. In this study, we investigated the effects of skin precursor cell-derived Schwann cells (SKP-SCs) directly cocultured with 6-OHDA-injured dopaminergic neurons in vitro and of SKP-SCs transplanted into the brains of 6-OHDA-induced PD mice in vivo. In vitro and in vivo studies revealed that SKP-SCs could reduce the damage to dopaminergic neurons by enhancing self-autophagy and modulating neuronal autophagy. Thus, the present study provides the first evidence that cell transplantation mitigates 6-OHDA-induced damage to dopaminergic neurons by enhancing self-autophagy, suggesting that earlier transplantation of Schwann cells might help alleviate the loss of dopaminergic neurons.

## Introduction

Parkinson’s disease (PD) is a neurodegenerative disease that is principally defined by the motor symptoms of resting tremor, rigidity, and bradykinesia. These symptoms are basically the result of the progressive loss of dopaminergic neurons in the substantia nigra (SN) pars compacta [1]. A longstanding hypothesis explaining the vulnerability of this cell population argues that dopamine induces neuronal injury via oxidative stress [2, 3]. Recent studies of PD have shown that dopaminergic neurons are susceptible to oxidative stress due to their inherent biological features [4, 5]. Dopamine oxidation generates electron-deficient quinones and reactive oxygen species, which can induce cellular dysfunction [6, 7]. Studies have reported that oxidatively modified proteins, lipids, and nucleic acids are deposited in the SN in the postmortem brain of patients with PD [8–10].

Autophagy is essential for survival, differentiation, development, and homeostasis, particularly [11] in response to oxidative stress [11–13]. This lysosomal degradation pathway controls the turnover of cytoplasmic contents and organelles by engulfing cargo into bilayer autophagosomes. The autophagy pathway is characterized by an increase in microtubule-associated protein 1 light chain 3 b-II and a decrease in sequestosome-1 (SQSTM1/p62) [14]. Accumulating evidence indicates that autophagy also occurs in PD and scavenges stress-damaged cell contents [15]. Autophagosome accumulation is evident in the brain tissues of 1-methyl-4-phenyl-1,2,3,6-tetrahydropyridine (MPTP)-induced PD animal models and patients with PD [16, 17]. However, excessive autophagy promotes programmed cell death, which is known as autophagic cell death or type II programmed cell death [18, 19]. The excessive upregulation of autophagy and long-term autophagic upregulation might eventually lead to self-digestion or exert damaging effects [20, 21]. Autophagy inhibition increases neuronal survival in conditions such as ischemic brain injury in mice and *Caenorhabditis elegans* cell necrosis [22–24].

Cell transplantation is gaining attention as an emerging treatment for PD [25]. Schwann cells (SCs) have been used to treat multiple cases of spinal cord injury [26–29]. For PD treatment, many animal studies have confirmed that SC transplantation effectively protects dopaminergic neurons in the brain or promotes the survival of cotransplanted dopamine-producing cells [30–33]. SCs generated from skin-derived precursors (SKP-SCs) compared with autologous cells after self-injury can better promote the regeneration of peripheral nerves, and functional recovery after injury also exhibits significant repair capacity for spinal cord injury [34–36].

Additionally, oxidative stress and autophagy are widespread in transplantation therapy and affect both graft survival and treatment outcome [37, 38]. Nevertheless, these data are mostly based on organ transplantation, such as kidney and liver transplantation, and stem cell transplantation, and the mechanism of SC transplantation for PD remains unclear. In contrast, clinical trials have found that pathological alpha-synuclein from patients with PD is deposited in peripheral nervous system SCs, which contribute to the recovery of the motor function of patients with PD when transplanted into the brain [39, 40]. These results demonstrate the complex role of SCs in the process of PD, and they indicate the need to further clarify their impact on dopaminergic neurons.

We hypothesized that SKP-SCs alleviate the damage to dopaminergic neurons caused by 6-OHDA-induced oxidative stress in neurons, which causes autophagy in neurons, and this sustained autophagy might lead to neuronal death. SKP-SCs affect autophagy in neurons and prevent neuronal death by enhancing self-autophagy. In this study, we verified our hypothesis using primary mouse mesencephalic neurons, retinoic acid-differentiated SH-SY5Y (RA-SY5Y) cells and mice, and we proposed the mechanism through which SKP-SCs protect dopaminergic neurons.

## Results

### SKP-SCs alleviated dopaminergic nerve injury in PD mice

**Fig. 1:**
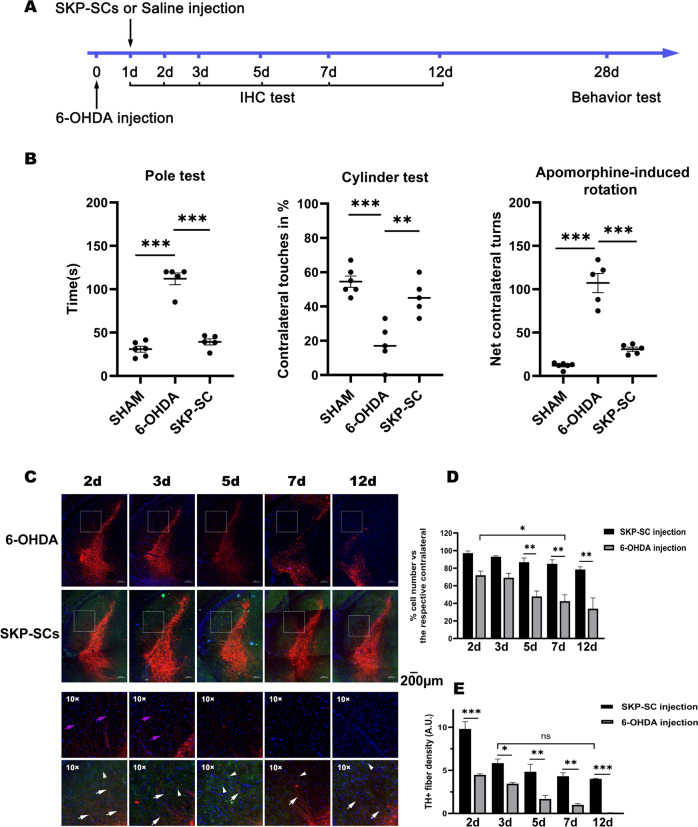
Comparison of behaviors and histofluorescence of PD mice after SKP-SC transplantation. **A** Animal experimental design. Animals were divided into a 6-OHDA injection group and a SKP-SCs injection group. Animals in both groups were sacrificed at 2, 3, 5, 7, and 12 days after injury, and immunohistochemical staining was performed. One month later, the behavior of the animals in both groups as well as that of those in the sham-operated group were tested. **B** All the results from behavioral tests, pole tests, cylinder tests, and apomorphine-induced totals suggested that the motor ability of SKP-SC-transplanted mice recovered better than that of 6-OHDA-injured mice. **C**–**E** Comparison of dopaminergic neuron numbers and fiber densities in the nigrostriatal sections of mice. A sustained decrease of 40.6% in the number of dopaminergic neurons was also observed on day 7 compared with the level at day 2. SKP-SCs transplantation alleviated this effect, as demonstrated by 55.7% increases compared with the levels in the 6-OHDA group at day 12. On day 2, the TH(+) fibers density in the SKP-SCs transplantation group was 2.2 times higher than that in the injury group. On day 3, there was a decrease in TH(+) fibers in the cell transplantation group (about 40.5%), but this decrease was not consistently worse, and the TH(+) fibers in the cell transplantation group were consistently significantly higher than those in the injury group from day 3 to day 12. TH(+) fibers (white arrow) and SKP-SCs (white arrowhead). **p ≤ 0.01, ***p ≤ 0.001. 6-OHDA 6-hydroxidopamine, TH tyrosine hydroxylase, SKP-SCs SCs generated from skin-derived precursors.

Our previous study found that the conditioned medium of SKP-SCs can alleviate the damage to SY5Y neuronal cells induced by 6-OHDA [41]. In the present study, we intended to investigate the effects of SKP-SCs directly transplanted into the brains of PD mice induced by 6-OHDA. The aim of this study tried to investigate whether SKP-SCs transplantation at early stage of PD could delay the disease progression and further to observe the effects for mice behavior after SKP-SCs transplantation for 1 month. We performed unilateral injections of SKP-SCs 1 day after 6-OHDA injection and assayed the behavioral ability of the mice 1 month after cell injection (Fig. 1A). All three behavioral tests showed that SKP-SC transplantation significantly improved the motor ability of PD mice (Fig. 1B). We then analyzed the number of dopaminergic neurons and the fiber density in the SN of mice at 2 day, 3 day, 5 day, 7 day, and 12 day after 6-OHDA injection. Immunohistochemical fluorescence showed that the number of dopaminergic neurons was significantly decreased on day 7, whereas the SKP-SCs-injected mice exhibited only a mild decrease in the dopaminergic neuron number (Fig. 1C, D). At day 2 postinjury (1 day after cell injection), the TH-positive fiber density in the SKP-SCs-injected group was significantly better than that in the 6-OHDA-injured group, but a subsequent decrease in nerve fiber density was also observed. Although the SKP-SCs injection group also showed a decrease in nerve fibers, there was no further decrease in nerve fibers at 12 d. In contrast, the nerve fibers in the injury group showed a continuous decrease (Fig. 1C, E).

The above results revealed that the early transplantation of SKP-SCs into 6-OHDA-induced PD mice resulted in the retainment of more intact dopaminergic neurons, which significantly improved the behavioral ability of the mice. However, the mechanism for the protective effect of SKP-SCs remained unknown. Because 6-OHDA damages neurons mainly through oxidative stress, autophagy is an essential response to oxidative stress. Current studies also suggest that neuronal autophagy is involved in neurodegenerative diseases, but the changes in autophagy induced by SC transplantation are unknown. Therefore, we further investigated the changes in autophagy during treatment with SKP-SC transplantation.

### SKP-SCs enhanced self-autophagy to alleviate excessive autophagy of dopaminergic neurons in vitro

**Fig. 2:**
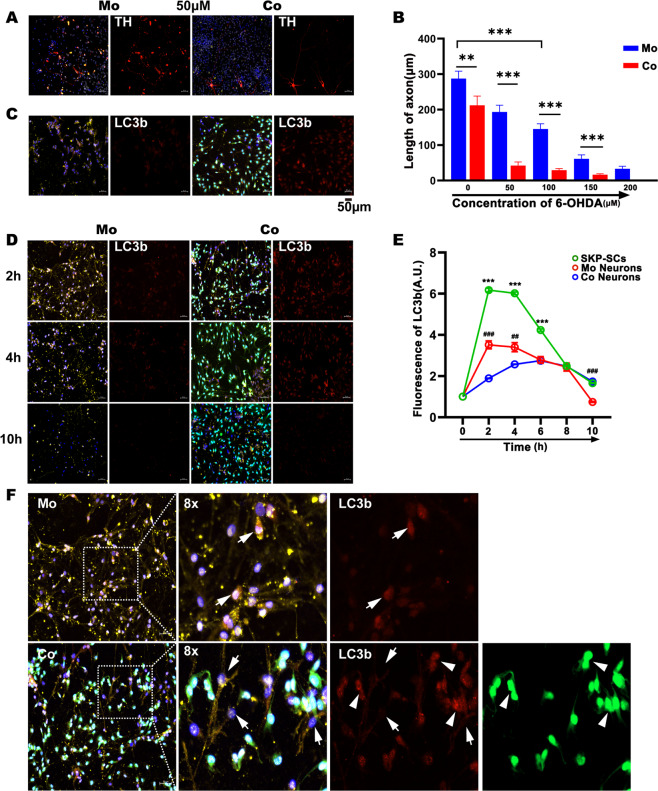
Alterations in the axon length and LC3b expression with or without coculture with SKP-SCs. **A**, **B** The direct coculture of SKP-SCs with mesencephalic neurons enhanced the resistance of neurons to different concentrations of 6-OHDA, and at 50 μM, the axons of monocultured neurons showed significant breakage, as demonstrated by an 82.1% reduction in length compared with the control group and a 79.8% reduction in the axon length compared with the coculture group. **C** At 6 h, LC3b of neurons and SKP-SCs under drug injury at 50 µM. It can be seen that some neurons in the coculture group retained axonal morphology. **D**, **E** Comparison of LC3b fluorescence between the two groups. The LC3b fluorescence in the monoculture group was significantly increased 2 h after injury and then gradually decreased, and by 10 h, a large number of neurons appeared dead. The fluorescence in the coculture group level increased to a 2.3-fold higher level compared with that of the monoculture group and then gradually decreased, and by 10 h, the neurons and SKP-SCs were alive. The LC3b fluorescence of each cell type in the coculture was analyzed separately, and the results suggested that in the coculture, the SKP-SCs exhibited a significant increase in the LC3b fluorescence at 2 h after injury to a level that was 3.3-fold higher than that obtained with the cocultured neurons and then decreased gradually; the LC3b fluorescence of the neurons in the coculture decreased after a mild and slow increase. Hash indicates the comparison of the LC3b fluorescence between neurons in the monoculture and coculture groups; Asterisk indicates the comparison of the LC3b fluorescence between SKP-SCs in the coculture group and neurons in the same group. **F** Local graphs of LC3b fluorescence in both groups at 2 h. Neurons (white arrows) and SKP-SCs (white arrowhead). **p ≤ 0.01, ***p ≤ 0.001, *N* = 3 per group.

We first observed the effects of different concentrations of 6-OHDA on axonal length in monocultures and cocultures with SKP-SCs. The results shown in Supplementary Fig. 2 demonstrate that neuronal axons grew longer in the coculture system [42]. The monocultured dopaminergic neurons exhibited significant axon breaks after stimulation with 50 μM 6-OHDA. Treatment with 100 μM 6-OHDA showed significant axon breaks, but the residual axon lengths were markedly longer than those in the corresponding monocultured neurons (Fig. 2A, B, Supplementary Fig. 2). We further observed the autophagy of neurons in both groups under 50 μM 6-OHDA, and the results showed that autophagy occurred in both groups at this concentration, and the axons of monocultured neurons disappeared, while some neurons in the coculture group still had some length of axon morphology (Fig. 2C).

To further explore the autophagy changes in direct coculture, we performed immunocytochemistry fluorescence analyses of both groups of cells after stimulation with 50 μM 6-OHDA. As expected, the neurons in the monoculture group showed a rapid increase in LC3b fluorescence after injury and a decrease in fluorescence while maintaining a high level of autophagy for some time (Fig. 2D-Mo, Supplementary Fig. 3A). This decrease was not due to the clearance of stress injury via the self-autophagy of neurons, but it was the result of the massive death of neurons under excessive autophagy. Many neurons died as determined using fluorescence and white light microscopy, and this finding was corroborated using a CCK8 assay of monocultured neurons (Supplementary Figs. 3C and 4A). Interestingly, the LC3b fluorescence of the coculture group also showed a rapid increase in fluorescence after injury, and the fluorescence intensity was markedly higher than that of the corresponding monocultured neurons. The fluorescence then gradually decreased to a lower level. Unlike the monocultured neurons, the neurons and SKP-SCs in the coculture system remained alive for 10 h after injury (Fig. 2D-Co, Supplementary Fig. 3B). To elucidate the mechanism of this phenomenon, the LC3b fluorescence of the two types of cells in the coculture was analyzed separately. The LC3b level in SKP-SCs increased rapidly after injury and was maintained for several hours before gradually decreasing to the LC3b level of the neurons in the coculture, whereas the LC3b of the neurons showed a mild and slow increase after injury and then decreased (Fig. 2E). Further observations revealed that the monocultured neurons showed significant LC3b fluorescence 2 h after injury. In the corresponding coculture system, SKP-SCs exhibited significant LC3b fluorescence, whereas their neighboring neurons showed lower LC3b fluorescence (Fig. 2F).

We also used the RA-SY5Y cells as an additional neuronal model to validate the effects induced by SKP-SCs in midbrain neurons. Here, RA-SY5Y was used as a model for PD studies because of its significant dopaminergic neuronal profile [43–45]. As a result, similar to the primary midbrain neurons, the LC3b fluorescence of RA-SY5Y cells in monoculture showed a rapid increase after 6-OHDA injury and then gradually decreased, and this decrease in fluorescence was due to cell death. In addition, SKP-SCs in coculture also showed a rapid increase in LC3b fluorescence, whereas the corresponding RA-SY5Y cells showed only a mild LC3b increase. Ten hours after injury, all the cells in the coculture survived (Supplementary Figs. 4B and 5).

### SKP-SCs reduced neuronal autophagy, and activated mTOR and AMPK were implicated in the autophagy of RA-SY5Y cells

**Fig. 3:**
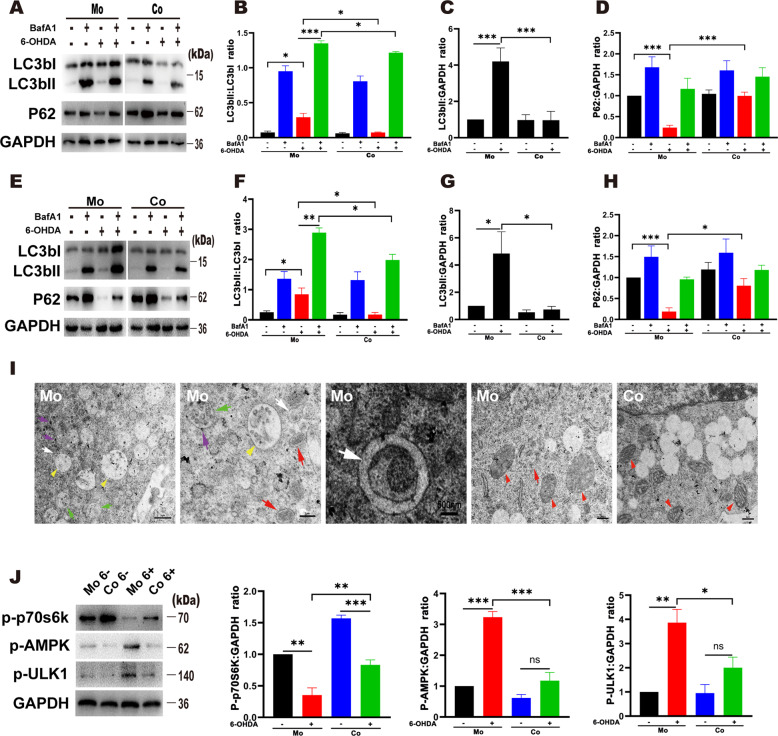
SKP-SC coculture altered autophagy in primary mesencephalic neurons and RA-SY5Y cells. **A**–**D** 6-OHDA induced a significant increase in LC3b in monocultured neurons, and this increase was further increased by the addition of bafilomycin; in contrast, P62 exhibited a decrease. The LC3b in the cocultured neurons was significantly lower than that in the monocultured group, the LC3b in the cocultured neurons was lower than that in the monocultured neurons after the addition of bafilomycin, and the P62 in the cocultured neurons was also higher than that in the monocultured neurons. **E**–**H** Immunoblot of RA-SY5Y cells. Both groups of RA-SY5Y cells showed a consistent trend with neurons after the same treatment. **I** Transmission electron micrographs of monocultured RA-SY5Y cells and cocultured cells. Two hours after injury, obvious lysosomes (purple arrow), autophagosomes (white arrow) and autolysosomes (red arrow) were detected in the monocultured cells. Multiple multivesicular bodies (yellow arrowhead) and amphisomes (green arrow) were produced, mitochondria (red arrowhead) showed obvious swelling, and mitochondrial cristae disappeared. In contrast, the SKP-SC coculture group exhibited a good mitochondrial morphology and did not show many multivesicular bodies or autophagosomes. **J** mTOR and AMPK were activated in RA-SY5Y cells in the monoculture group. The level of p-p70s6k was significantly decreased and the levels of p-AMPK and p-ULK1 were significantly increased in the monoculture group. The level of p-p70s6k was decreased in the coculture group but was still higher than that in the monoculture group; p-AMPK and p-ULK1 were not significantly elevated. *p ≤ 0.05, **p ≤ 0.01, ***p ≤ 0.001. RA-SY5Y retinoic acid-differentiated SH-SYSY cells, BafA1 bafilomycin A1.

Western blot analysis confirmed that 6-OHDA enhanced the autophagy of neurons. To further observe the changes in autophagic flow, we used bafilomycin, which inhibits acidification inside the lysosome and blocks fusion between the autophagosome and the lysosome. The results showed a significant increase in LC3b in neurons after treatment with bafilomycin, suggesting a sustained increase in autophagic flow in mesencephalic neurons with 6-OHDA-induced injury. In contrast, western blotting of isolated neurons in the SKP-SCs group showed low LC3b levels and low autophagic flow (Fig. 3A–C). Moreover, P62 expression in neurons in the monoculture group decreased significantly after injury, whereas P62 expression in neurons in the coculture group remained almost unchanged after injury but showed differences from the P62 levels in monoculture neurons (Fig. 3A, D). Overall, these results suggested that SKP-SCs affected neuronal autophagy and avoided neuronal death due to excessive autophagy induced by 6-OHDA. In the coculture western blotting experiments with RA-SY5Y, the findings were consistent with those obtained for primary mesencephalic neurons (Fig. 3E–H).

To further clarify the autophagy process of the two groups of cells, we analyzed the two groups of cells at 2 h using transmission electron microscopy (TEM). The results showed that the monocultured RA-5Y5Y cells had many multivesicular bodies and amphisomes as well as increased numbers of lysosomes, autophagosomes and autolysosomes 2 h after 6-OHDA injury, and these effects were accompanied by mitochondrial swelling and the disappearance of mitochondrial ridges (Fig. 3I-Mo). In contrast, no significant increases in autophagosomes were detected in the cocultured RA-SY5Y cells, and the mitochondrial morphology remained normal (Fig. 3I-Co).

To further understand the mechanism through which SKP-SCs affect RA-SY5Y autophagy, we analyzed the western blots of isolated RA-SY5Y cells and found that both mTOR and AMPK were involved in 6-OHDA-induced RA-SY5Y autophagy (Fig. 3J). Because the inhibitory function of mTOR in autophagy has been well established [14], the phosphorylation of p70S6K at Thr389 by mTOR was also evaluated to assess mTOR activity. We found that monocultured RA-SY5Y cells showed a significant decrease in p70S6K phosphorylation, an increase in AMPK phosphorylation, and an increase in downstream ULK1 phosphorylation in response to 6-OHDA injury. This result suggested that the inhibition of autophagy by mTOR was decreased in RA-SY5Y cells under 6-OHDA injury and that mitochondrial injury induced a disturbance in energy metabolism, which also resulted in the phosphorylation of AMPK and ultimately led to enhanced autophagy. In contrast, although the cocultured RA-SY5Y cells showed a decrease in p70S6K phosphorylation, this decrease was lower than that found in RA-SY5Y cells in the monoculture group and was not accompanied by significant AMPK phosphorylation or significant ULK1 phosphorylation; ultimately, no intense autophagy was detected. In conclusion, SKP-SCs alleviated 6-OHDA-induced autophagy in RA-SY5Y cells, and the results implicated mTOR and AMPK activation in this process.

### SKP-SCs enhanced self-autophagy to alleviate excessive autophagy in dopaminergic neurons in vivo

The in vitro experiments revealed that SKP-SCs enhanced the resistance of neurons to 6-OHDA-induced oxidative stress damage by enhancing self-autophagy and alleviating the excessive autophagy of cocultured neurons. Given the protective effect of SKP-SCs on dopaminergic neurons in PD mice after transplantation of the cells into the brain, we investigated whether SKP-SCs also underwent similar changes in vivo to affect autophagy in dopaminergic neurons.

**Fig. 4:**
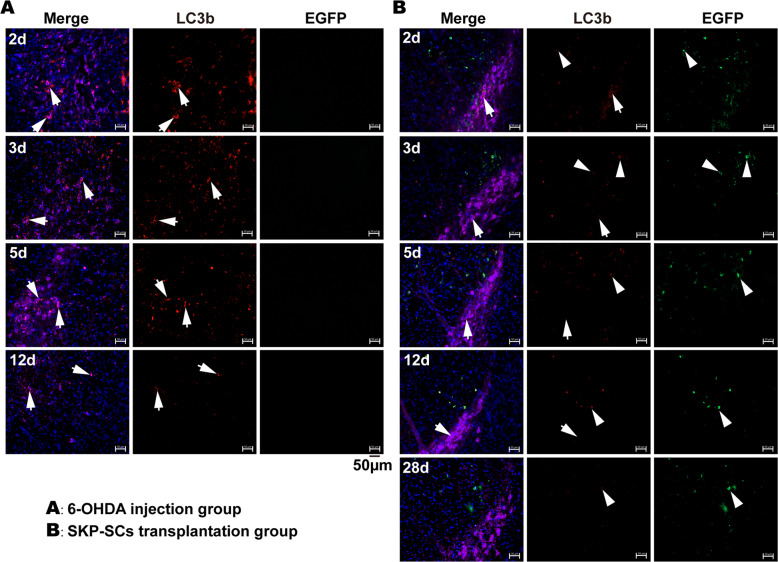
Autophagy in SKP-SCs and dopaminergic neurons in vivo. **A** From day 2 to day 5, most dopaminergic neurons(in purple) in the substantia nigra of mice in the injury group showed significant LC3b fluorescence, and most dopaminergic neurons were lost at day 12, with significant LC3b fluorescence still present in the surviving dopaminergic neurons. **B** On day 2, the nigrostriatal dopaminergic neurons in the SKP-SCs transplantation group showed some LC3b fluorescence, and from day 3, dopaminergic neurons showed a gradual decrease in LC3b fluorescence. By day 28, only a few dopaminergic neurons had a slight LC3b fluorescence. After transplantation into the brain within 12 d, SKP-SCs showed relatively significant LC3b fluorescence, and some SKP-SCs also showed a decrease in LC3b fluorescence at day 28. Dopaminergic neurons (white arrows) and SKP-SCs (white arrowhead).

The staining of brain sections showed significant LC3b fluorescence in the nigrostriatal dopaminergic neurons of 6-OHDA-injured mice, and most of the dopaminergic neurons showed persistently high levels of autophagy until neuronal death after day 2 to day 12 postinjury (Fig. 4A). In the SKP-SCs injection group, most of the dopaminergic neurons also showed some degree of autophagy at day 2, but only some dopaminergic neurons showed low levels of autophagy from day 3 to day 12, and most of the dopaminergic neurons did not show significant autophagic fluorescence. From day 2 to day 12, SKP-SCs showed consistently high levels of autophagy. We further observed the brain sections of mice in the day 28 cell injection group. In this group, only a few dopaminergic neurons showed low levels of autophagic fluorescence, while some SKP-SCs also showed decreased autophagic fluorescence (Fig. 4B).

## Discussion

**Fig. 5:**
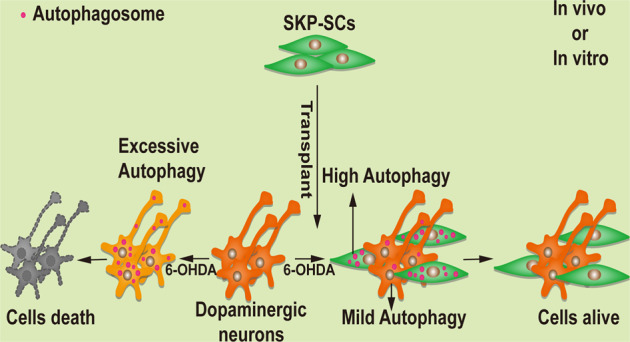
Mechanism of protection on dopaminergic neurons by SKP-SCs. SKP-SCs enhanced self-autophagy to alleviate excessive autophagy in dopaminergic neurons induced by oxidative stress in vitro and vivo, enhancing cell survival.

Activation of autophagy has been observed in 6-OHDA-induced PD models, and modulation of oxidative stress-induced excessive autophagy on dopaminergic neuronal damage is a promising strategy for the treatment of PD [46]. In the present study, SKP-SCs transplanted into PD mice promoted motor recovery ability. Immunofluorescence showed that SKP-SCs improved the ability of dopaminergic neurons to resist 6-OHDA-induced oxidative stress injury [47], promoted the survival of dopaminergic neurons, and maintained a higher fiber density. Further studies revealed that SKP-SCs affected the autophagy of cocultured neurons or RA-SY5Y cells in vitro by enhancing self-autophagy, avoiding excessive autophagy induced by oxidative stress, and enhancing cell survival. We subsequently found a similar situation in vivo. It should be noted that the fluorescence assay of brain sections from SKP-SC-injected mice on day 28 showed reduced autophagic fluorescence in some SKP-SCs accompanied by slight autophagy in a few dopaminergic neurons, which may be due to the gradual clearance of stress damage caused by 6-OHDA. The excessive autophagy of neurons might lead to axonal damage, which further causes neuronal death, and the moderate autophagy of neurons after SKP-SC transplantation might facilitate the clearance of stress-induced damage without causing autophagic death, which was consistent with the phenomena observed in the in vitro and in vivo experiments. Notably, the relatively high level of autophagy presented by most dopaminergic neurons in the SKP-SCs-injected group of mice on day 2 may be related to the subsequent neuronal axonal damage. This finding provides the first preliminary insight into changes in the autophagy of SCs and dopaminergic neurons in PD mice after transplantation therapy (Fig. 5).

The survival and function of transplanted cells in the recipient are keys to the success of the transplantation treatment. Researchers have found that oxidative stress and autophagy exist during transplantation, and pretreatment of stem cells to increase autophagy can enhance posttransplantation survival and function [48]. Moreover, the efficacy of cell transplantation is not limited to cell replacement but also includes modification of the pathological environment of the recipient neurons by the transplanted cells, which provides various trophic factors, reduces inflammatory damage, and effectively promotes the survival of surrounding neurons [49–51]. In the present study, SKP-SCs cocultured with neurons or RA-SY5Y cells exhibited higher levels of self-autophagy after 6-OHDA injury and were maintained until the neuronal or RA-SY5Y cell autophagy levels returned to normal. In particular, significant autophagy was also observed in transplanted SKP-SCs in vivo, which was in contrast to the dopaminergic neurons with low autophagy. High levels of autophagy in SKP-SCs in vivo might result from environmental changes after transplantation or 6-OHDA injury. Regardless, the ultimate result is the survival of SKP-SCs and the protection of surrounding neurons. The highly autophagic state of SCs might activate C-JUN to produce a series of secretory proteins, including glial cell line-derived neurotrophic factor, artemin, brain-derived neurotrophic factor, neurotrophin-3, nerve growth factor, tumor necrosis factor *α*, interleukin-1, and leukemia inhibitory factor [52, 53]. These trophic factors and inflammatory factors might provide nutritional support to injured neurons and reduce the local inflammatory response. Conditioned medium of some stem cells is thought to be neuroprotective [54], and our previous study also demonstrated that conditioned medium of SKP-SCs alleviates 6-OHDA damage to SY5Y cells in vitro [41]. However, most of these studies are based on the possible secretion of cytokines by cells and cannot simulate the growth of transplanted cells in a nascent environment or the interactions between the implanted cells and the recipient cells because these changes affect cell growth and secretion. Therefore, we obtained direct observations of cellular interactions, and the results provide an intuitive theoretical basis for cell therapy.

Dopaminergic neurons are vulnerable to oxidative stress [3], and a homeostatic mechanism in response to oxidative stress is necessary. In contrast, cells under sustained oxidative stress often show excessive autophagy, which eventually leads to cell death. Both cultured primary mesencephalic neurons and RA-SY5Y cells exhibited excessive autophagy in response to 6-OHDA injury, which resulted in cell death, and numerous autophagy-associated structures, such as autophagosomes, multivesicular bodies, and amphisomes, accompanied by mitochondrial damage were observed in the injured cells [55], suggesting that oxidative stress damage might also affect the energy metabolism of the cells while inducing autophagy [56]. Western blotting also indicated that mTOR and AMPK activation is involved in autophagy in monocultured RA-SY5Y cells. mTOR is a core component in a series of autophagy signaling networks, including induction, progression, and autophagy termination. Additionally, mTOR senses different stimuli, such as insulin, energy, and amino acid levels [57]. Previous studies have found that 6-OHDA affects the mTOR phosphorylation level in SY5Y cells or animals [58, 59]. Our experiments revealed that 6-OHDA also caused a decrease in mTOR phosphorylation in RA-SY5Y cells. Although autophagy can remove the contents of cells damaged by oxidative stress and provide an energy supply, the stages of autophagy require energy support [60], and damaged mitochondria fail to provide a similar support and further induce AMPK activation and aggravate autophagy [61].

RA-SY5Y cells in the coculture group also exhibited mTOR activation, which indicated that 6-OHDA caused oxidative stress and autophagy in RA-SY5Y cells in the coculture system, whereas AMPK was not activated in RA-SY5Y cells, which suggested that the energy metabolism in the cells was normal. AMPK in cocultured RA-SY5Y cells was not activated, which suggested that the energy metabolism of the cells was as expected, and TEM observations confirmed that the morphology of the mitochondria was good and that the cells met the energy requirements of autophagy. The appropriate intensity of autophagy maintained cell survival after the removal of 6-OHDA-induced damage.

In response to oxidative stress, the timing and degree of autophagy are critical. Some studies have suggested that enhanced autophagy might effectively clear abnormal proteins in neurodegenerative diseases with abnormal protein accumulation, such as PD [62]. However, for stress injuries, excessive autophagy might be counterproductive [63]. Furthermore, dopamine oxidation also promotes abnormal accumulation of α-syn^3^. Modulating the level of abnormal autophagy at the early stages of the disease and reducing the damage caused by oxidative stress might also reduce the accumulation of abnormal proteins. In the present study, SKP-SCs affected the excessive activation of neuronal autophagy caused by oxidative stress by enhancing self-autophagy such that neuronal autophagy became mild and long lasting, resulting in the clearance of oxidative stress damage and the avoidance of neuronal death. Therefore, earlier intervention for dealing with oxidative stress may result in a better outcome. For example, in the trial of DBS combined with autologous sural nerve grafting in patients with PD conducted by Van et al., DBS was usually performed in patients with motor disorders with a long disease duration and who did not respond well to pharmacological treatment. According to our experiments, it is possible that the early transplantation of SCs will lead to better clinical outcomes.

## Materials and methods

### Cell culture

The morphology of the three types of cells in the monocultures and cocultures is shown in Supplementary Fig. 1. The details of the experiments are detailed below.

### RA-SY5Y

The SH-SY5Y neuroblastoma cell line was purchased from ATCC. Cells were grown in Dulbecco’s modified Eagle’s medium/nutrient mixture F-12 (DMEM/F12; Thermo Fisher Scientific, 11320–033), 2 mM L-glutamine (Thermo Fisher Scientific, 25030081), 100 U/mL penicillin, 100 μg/mL streptomycin (Thermo Fisher Scientific, 15140122), and 10% fetal bovine serum (FBS; GIBCO, 16140071). Cells were maintained at 37 °C in a saturated humidity atmosphere containing 95% air and 5% CO_2_. The cellular differentiation protocol has been previously described [43, 64]. In brief, cells were seeded at an initial density of 10^4^ cells/cm^2^ in culture dishes with complete medium. Trans retinoic acid (RA; Sigma-Aldrich, PHR1187) was added the day after plating at a final concentration of 10 µM in DMEM/F12 with 3% FBS, and cells were incubated for 7 days. The medium was replaced every other day, and on day 7, cells were harvested and used for experiments.

### Primary dopaminergic neuron cultures

Primary dopaminergic neurons from embryonic ventral mesencephalic neurons were isolated as described previously [65]. Briefly, E13 pregnant SD rats were sacrificed by cervical dislocation, and the embryos were collected in Ca2-Mg2-free Hanks’ balanced salt solution. The ventral mesencephalic tissues were dissected, dissociated, and then treated with Accutane (Thermo Scientific, A1110501) for 8 min at 37 °C. After gentle blowing, the centrifuge tube was placed on ice and allowed to rest for 5 min before the upper cell suspension was aspirated; this operation was repeated three times before the sediment was discarded and the cell suspension was centrifuged at low speed. The pellet was resuspended in 2 mL of complete medium by slowly pipetting up and down ~20 times. Cells were plated on coverslips coated with poly-L-lysine solution (Sigma, P4832) and laminin (Sigma, L2020) in 24-well plates at a density of 150,000 cells/well and at a density of 2 million cells/dish in a coated 3.5-cm dish. Cells were maintained in complete medium for 4 h. The medium was then changed to neurobasal medium (Thermo Scientific, 21103049) plus B27 supplement (Stemcell, 05711), 2 mM GlutaMAX™ supplement (Thermo Scientific, 35050061), 100 U/mL penicillin, and 100 μg/mL streptomycin for 7 days. The medium was replaced every other day, and on day 7, cells were harvested and used for experiments.

### EGFP-SKP-SCs and cocultivation

EGFP-SKP-SCs were kindly provided by Dr. Chengbin Xue (Jiangsu Key Laboratory of Neuroregeneration, Nantong University) and have been successfully applied in a previous study [66, 67]. Cells were cultured in DMEM/F12 (3:1) medium supplemented with 1% FBS, 2% N2 supplement (Thermo Scientific, 17502048), 5 µM forskolin (R&D Systems, 1099), 50 ng/mL Heregulin-1β (R&D Systems, 396-HB), 100 U/mL penicillin, and 100 μg/mL streptomycin. When cells were ~80% confluent, they were digested with trypsin and used for coculture. For direct coculture, SKP-SCs were seeded at a density of 4 × 10^4^ cells/well to differentiate RA-SY5Y cells or primary dopaminergic neurons at a mixture of 1:1 with the respective medium in 24-well plates. SKP-SCs were also used for intracranial injections.

### Cell proliferation assay

A Cell Counting Kit-8 (Abcam, ab228554) was used to assess the viability of primary neurons. Neurons were seeded at a density of 5000 cells/well into 96-well plates, and they were incubated for 7 days at 37 °C in a 5% CO_2_ incubator. Subsequently, 10 µL of CCK-8 reagent was added to each well, and the plate was incubated for 1 h. The cell absorbance was measured at a wavelength of 450 nm.

### Flow cytometry cell sorting

The collected cocultured cells were sorted using a flow cytometer, and the EGFP-SKP-SCs in the coculture were self-fluorescent to facilitate sorting. The sorted RA-SY5Y cells and primary mesencephalic neurons were placed in phosphate-buffered saline (PBS) at 4 °C for other experiments.

### TEM ultrastructural morphological analysis

The sorted RA-SY5Y cells were fixed in phosphate-buffered 2% glutaraldehyde, postfixed in 2% osmium tetroxide for 3 h in an ice bath, dehydrated in ethanol, and embedded in epoxy resin. Ultrathin sections were obtained using the ultramicrotome technique. Ultrathin sections stained with uranyl acetate for 10 min and with lead staining solution for 5 min were subjected to TEM (HITACHI, HT7700) observations at the Key Laboratory of Neuroregeneration (Nantong University).

### Animal experiments

The animal experimental procedure is shown in Fig. 1A. The details of the procedure are described below.

### 6-OHDA lesion

All experimental protocols were performed following the guidelines on animal research provided by the Institutional Ethics Committee at Nantong University and were approved by the Ethics Committee. Adult C57BL/6 J male mice (25–30 g) were maintained under 12-h light/12-h dark cycles in cages and acclimated to the experimental environment for 1 week before modeling. Mice received a unilateral intrastriatal injection of 6-OHDA. Animals were pretreated with desipramine [68]. A total dose of 15 μg of 6-OHDA was infused into the right striatum at the following coordinates: anterior-posterior (AP), +0.09 cm; medial-lateral (ML), +0.22 cm; and dorsal-ventral (DV), −0.25 cm relative to the bregma.

### Cell transplantation

Mice were randomly assigned to the following two groups: (1) the SKP-SC-treated group (6-OHDA + SKP-SCs) and (2) the control group (6-OHDA + vehicle). Mice received unilateral intrastriatal transplantation of SKP-SCs or vehicle the days after 6-OHDA administration. The burr hole was formed unilaterally at the following coordinates: anterior-posterior (AP), +0.06 cm; and medial-lateral (ML), +0.20 cm relative to bregma. Each animal in the treated group received 1 μL of the cell suspension at the following coordinates: dorsoventral, −0.22 and −0.28 cm, relative to the dura. Approximately 6 × 10^4^ SKP-SCs in 0.5 μL of PBS or an equal volume of vehicle were transplanted at two sites via a microsyringe at an infusion rate of 0.1 μL/min to obtain a total dose of 1.2 × 10^5^/μL. The needle was withdrawn slowly following a wait time of 2 min. The animals were returned to a temperature-controlled blanket until they recovered from the anesthesia.

### Behavioral testing

All tests were performed 4 weeks after 6-OHDA injection. In the pole test, the mice were placed head-upward on top of a rough-surfaced iron pole (50 cm in length and 1.0 cm in diameter), and the mice could climb down to the base of the pole. The time that was required for each mouse to turn completely downward and then reach the floor was measured with a cutoff of 120 s. The average of the three measurements was used as the result. In the cylinder test, each mouse was placed into a transparent cylinder (25 cm in diameter and 40 cm in height) for 3 min, and the frequencies at which the left forelimb, right forelimb, and both forelimbs were used during rearing were recorded by a blinded examiner. The data are presented as the frequency ratios of left to right + left forelimb wall touches. In the apomorphine-induced rotation test, the mice were allowed to habituate for 10 min in a white 30 × 30-cm chamber. After an intraperitoneal injection of 0.5 mg/kg apomorphine hydrochloride, the full rotations in the chamber were recorded with a video camera for 30 min and counted by a blinded examiner.

### Tissue preparation

Perfusion was performed with a cold saline solution, and fixation was then performed with 4% paraformaldehyde in 0.1 M phosphate buffer. Each brain was dissected, postfixed overnight in buffered 4% paraformaldehyde at 4 °C, and stored in a 30% sucrose solution at 4 °C until it sank. Frozen sectioning was performed on a freezing microtome (Leica, CM3050S) to generate 20-μm-thick coronal sections.

### Immunocytochemistry and immunohistochemistry

Cells in 24-well plates were fixed with 4% paraformaldehyde for 10 min at room temperature (RT), washed with PBS, permeabilized with 0.1% Triton X-100 for 10 min at RT, and treated with 10% goat serum blocking buffer for 30 min at RT. Primary neurons were stained with primary antibody against tyrosine hydroxylase (TH; 1:300, Abcam, ab137869) as a marker for dopaminergic neurons. Neurons were stained with an antibody against NeuN (1:1000, Abcam). Anti-LC3b (1:1000, ab192890) was used as an autophagy marker for neurons and RA-SY5Y cells, and βIII-tubulin (1:1000, Sigma, T5076) was used as a marker of cell morphology. Cells were incubated with the primary antibodies overnight at 4 °C. After washing, cells were incubated with secondary antibodies (Alexa Fluor 568-conjugated goat anti-rabbit or Alexa Fluor 647-conjugated goat anti-mouse; 1:1000 dilution) for 1 h at RT in the dark. Coverslips were then washed with PBST and treated with antifade mounting medium containing Hoechst 33342. The prepared tissue sections were treated according to a similar staining procedure, washed with washing solution containing TBS, and incubated with blocking buffer containing 3% BSA and 10% goat serum for 2 h at RT. Anti-TH(1:1000, Abcam, ab76442) was used to label TH-positive neurons, and cells were costained with LC3b. Cells were then incubated with secondary antibodies for 2 h at RT in the dark. The reaction was terminated as described above. Images were obtained using a microscope (Carl Zeiss, Axio Imager M2), and all images were acquired using the same exposure time. For immunocytochemistry, six to nine fields (two to three fields × three independent samples) were selected randomly from each group, and for immunohistochemistry, three sections from each animal (three mice) were randomly selected. The number of TH-positive neurons, the fractions of TH-positive areas in the SN, and the fluorescence intensity of LC3b in neurons and RA-SY5Y cells were measured using ImageJ Fiji 1.53c (National Institutes of Health, USA).

### Western blotting

Cells were homogenized in RIPA lysis buffer (EpiZyme, PC101), containing protease inhibitor cocktail (MCE, HY-K0010) and phosphatase inhibitor cocktail I (MCE, HY-K0021), and then centrifuged at 1600 × *g* and 4 °C for 20 min. Supernatants were collected, and protein concentrations were determined using a BCA Protein Assay Kit (Beyotime, P0012). An aliquot of the supernatant was diluted in SDS-PAGE Sample Loading Buffer (Beyotime, P0015L), and proteins were separated on Omni-PAGE™ HEPES-Tris Gels (EpiZyme, LK212) and transferred to a polyvinylidene difluoride membrane (Merck Millipore, P2938). The membrane was blocked for 1 h at RT in blocking buffer [TBS containing 5% Difco™ skim milk (Becton, Dickinson and Company, USA) and 0.1% Tween 20] and subsequently incubated with the following primary antibodies overnight at 4 °C: rabbit anti-GAPDH (1:10000, Abcam, ab181602), rabbit anti-TH (1:1000, ab137869), rabbit anti-SQSTM1/p62 (1:1000, ab207305), rabbit anti-phospho-AMPKα (Thr172) (1:1000, CST, 8208), rabbit anti-phospho-ULK1 (Ser555) (1:1000, CST, 97094), and rabbit anti-phospho-p70S6 kinase (1:1000, CST, 8209). The membrane was washed in TBST and incubated with goat anti-rabbit IgG (H + L) and cross-adsorbed secondary antibody (HRP) (Thermo Fisher, G-21234) for 1 h at RT. The membrane was then washed three times in TBST for 5 min. The antigen-antibody peroxidase complex was detected using the High-sig ECL Western Blotting Substrate (Tanon™, 180-5001) according to the manufacturer’s instructions, and images were obtained using the Tanon™ 5200CE Chemi-Image System. The intensity of each band was determined by ImageJ Fiji 1.53c.

### Statistical analysis

All data are presented as the means ± SEM and were analyzed using GraphPad Prism 8.0. The difference between two groups was analyzed by a two-tailed Student’s *t*-test, and one-way ANOVA followed by Tukey’s post hoc analysis was used for multiple comparisons among two or more groups. A *P* value < 0.05 was defined as the threshold for statistical significance.

## Supplementary information

Supplementary Figure and Table Legends

supplementary fig 1

supplementary fig 2

supplementary fig 3

supplementary fig 4

supplementary fig 5

Table_Revision
