# Supplementary material for: SKP-SCs transplantation alleviates 6-OHDA-induced dopaminergic neuronal injury by modulating autophagy
Source: Cell Death Dis. 2021 Jul 5;12(7):674. doi: 10.1038/s41419-021-03967-3 (PMC8257782; doi:10.1038/s41419-021-03967-3)
Supplement: Supplementary file 1 — Supplementary Figure and Table Legends [file 41419_2021_3967_MOESM1_ESM.docx]

**Supplementary Fig 1. The culture and morphology of various cells. (A) Culture of primary neurons and differentiation of RA-SY5Y cells and their respective direct coculture with SKP-SCs. (B)** Primary mesencephalic neurons cultured for 7 days. **(C)** Primary mesencephalic neurons and SKP-SCs cocultured for 24 h. **(D)** RA-SY5Y cells cultured in induced differentiation medium for 7 days. **(E)** RA-SY5Y and SKP-SCs cocultured for 24 h. **(F)** SKP-SCs cultured for 3 days. Abbreviations: RA-SY5Y, retinoic acid-differentiated SH-SYSY cells; SKP-SCs, SCs generated from skin-derived precursors. Abbreviations: CM, complete medium; RA, retinoic acid; 6-OHDA, 6-hydroxidopamine; TH, tyrosine hydroxylase; SKP-SCs, SCs generated from skin-derived precursors.

**Supplementary Fig 2.** **Axonal changes in monocultured primary mesencephalic neurons and cocultured neurons under different concentrations of 6-OHDA.** **(A)** Monoculture group. **(B)** Coculture group. SKP-SCs were seeded after 7 days of neuronal growth, and the culture was continued for 24 h. Different concentrations of 6-OHDA were then added, and 6 h later, cellular immunofluorescence was performed.

**Supplementary Fig 3. Autophagy of primary mesencephalic neurons in monoculture and coculture at different times under 6-OHDA injury. (A)** LC3b fluorescence of monocultured primary mesencephalic neurons. **(B)** LC3b fluorescence of primary mesencephalic neurons cocultured with SKP-SCs. **(C)** Statistical analysis of cell viability in monocultured neurons. A substantial decrease in viability was detected starting at 6 h postinjury, and the viability did not show a significant difference at 8 h to 10 h. The data are presented as the means ± SEM. ***p ≤ 0.001, ****p ≤ 0.0001.

**Supplementary Fig 4. Cell morphology at 10 h after 6-OHDA-induced injury.** **(A)** Morphology of primary mesencephalic neurons 10 h after injury. The majority of the cell bodies were pyknotic, and the axonal morphology was lost. **(B)** Morphology of RA-SY5Y cells 10 h after injury. Most of the cell bodies were pyknotic, and a few of the surviving cells exhibited shortened synapses.

**Supplementary Fig 5. Autophagy of RA-SY5Y cells in monoculture and coculture.** The LC3b fluorescence of monocultured RA-SY5Y cells was significantly higher at 2 h and the gradually decreased; at 10 h, most of the cells had died, and some cells with mild autophagy survived (white arrow). The LC3b fluorescence of cocultured RA-SY5Y cells was mildly elevated at 2-4 h (white arrow), and the surrounding SKP-SCs showed distinct LC3b fluorescence (white arrowhead); at 10 h, the fluorescence of RA-SY5Y cells had almost disappeared, and SKP-SCs with mild fluorescence were detected.

**Table: Antibodies and Materials.**
