# Supplementary figures and images for: SKP-SCs transplantation alleviates 6-OHDA-induced dopaminergic neuronal injury by modulating autophagy
Source: Cell Death Dis. 2021 Jul 5;12(7):674. doi: 10.1038/s41419-021-03967-3 (PMC8257782; doi:10.1038/s41419-021-03967-3)

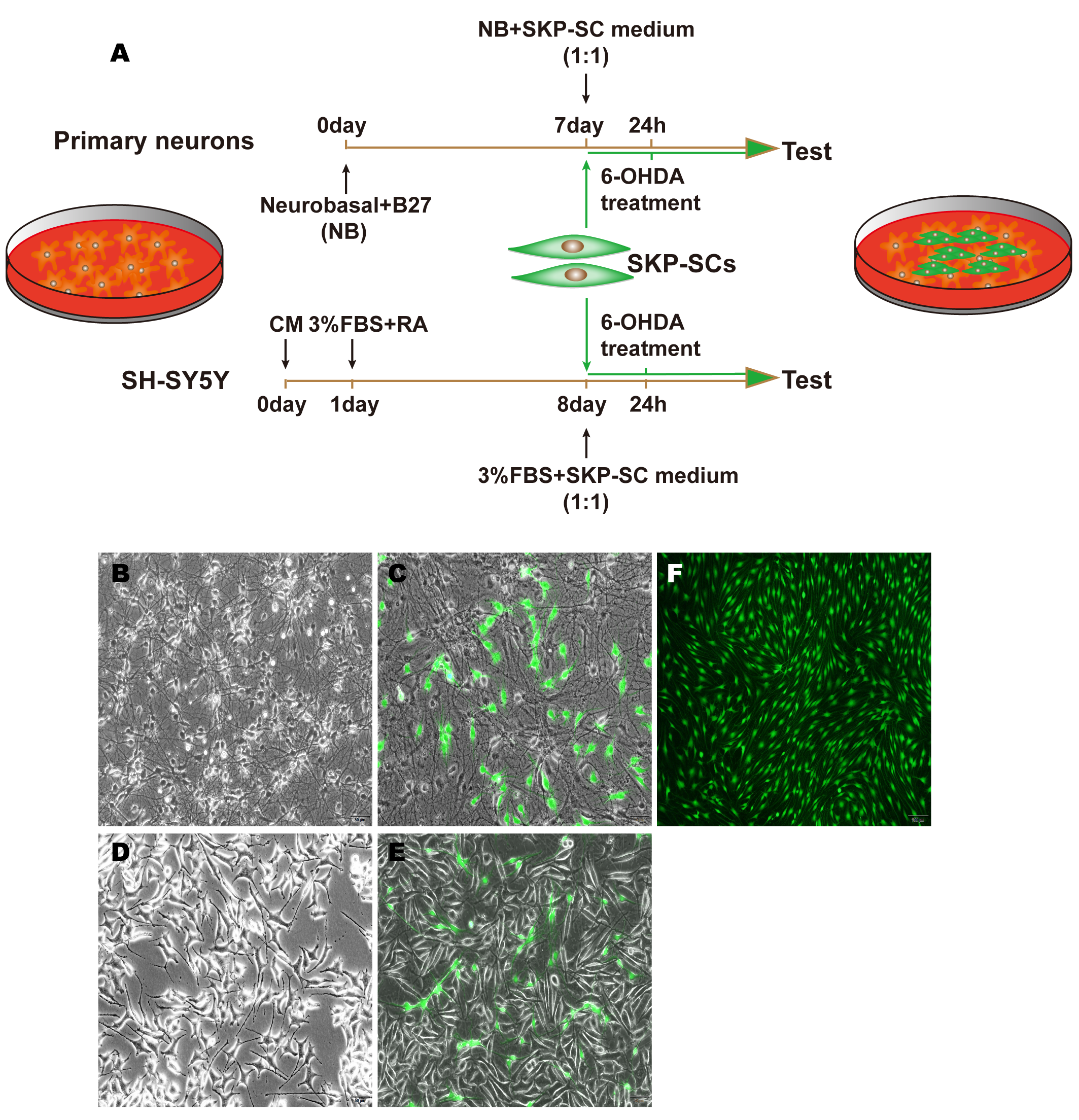

Supplement: Supplementary file 2 — supplementary fig 1 [file 41419_2021_3967_MOESM2_ESM.tif]

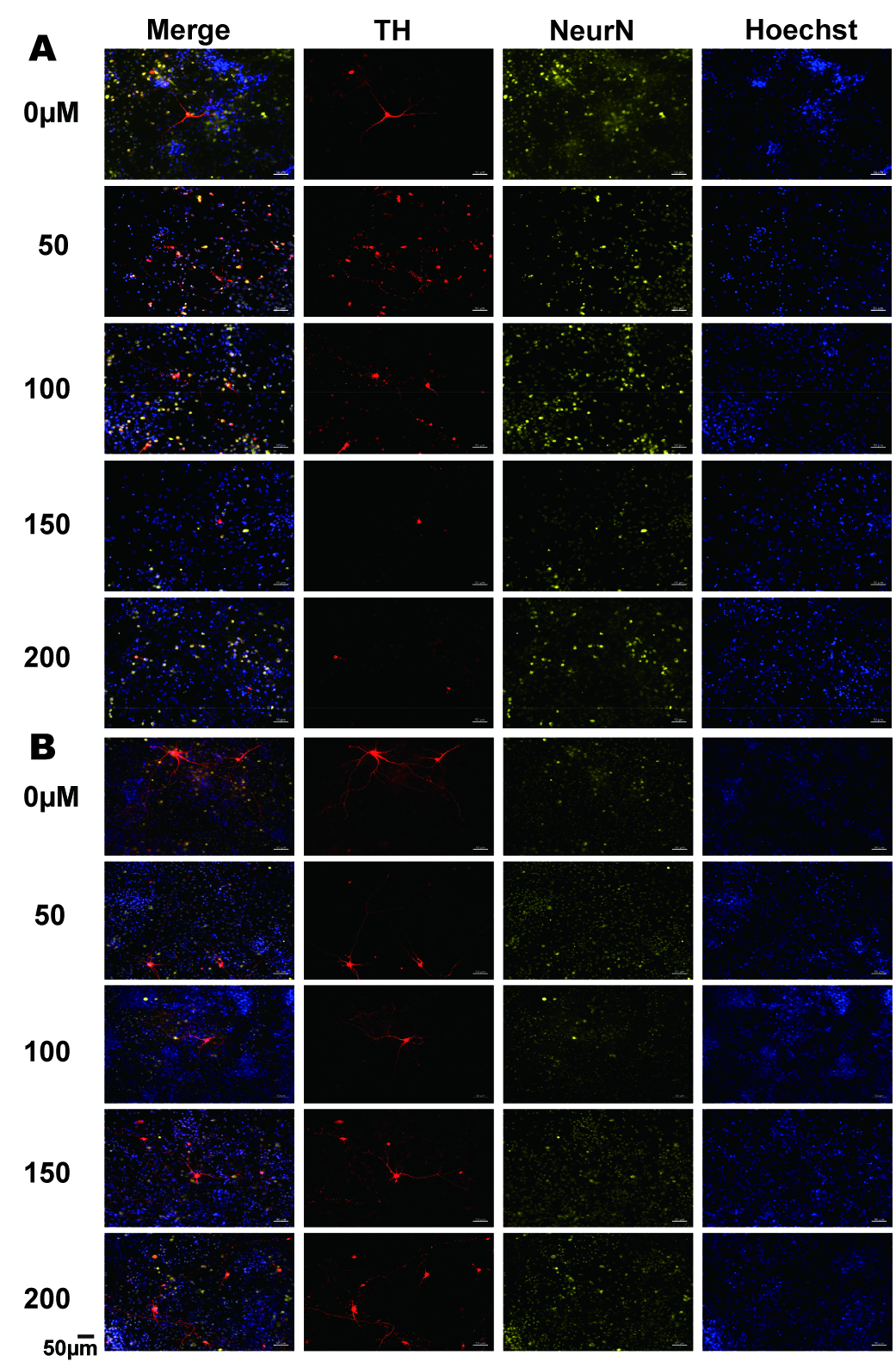

Supplement: Supplementary file 3 — supplementary fig 2 [file 41419_2021_3967_MOESM3_ESM.tif]

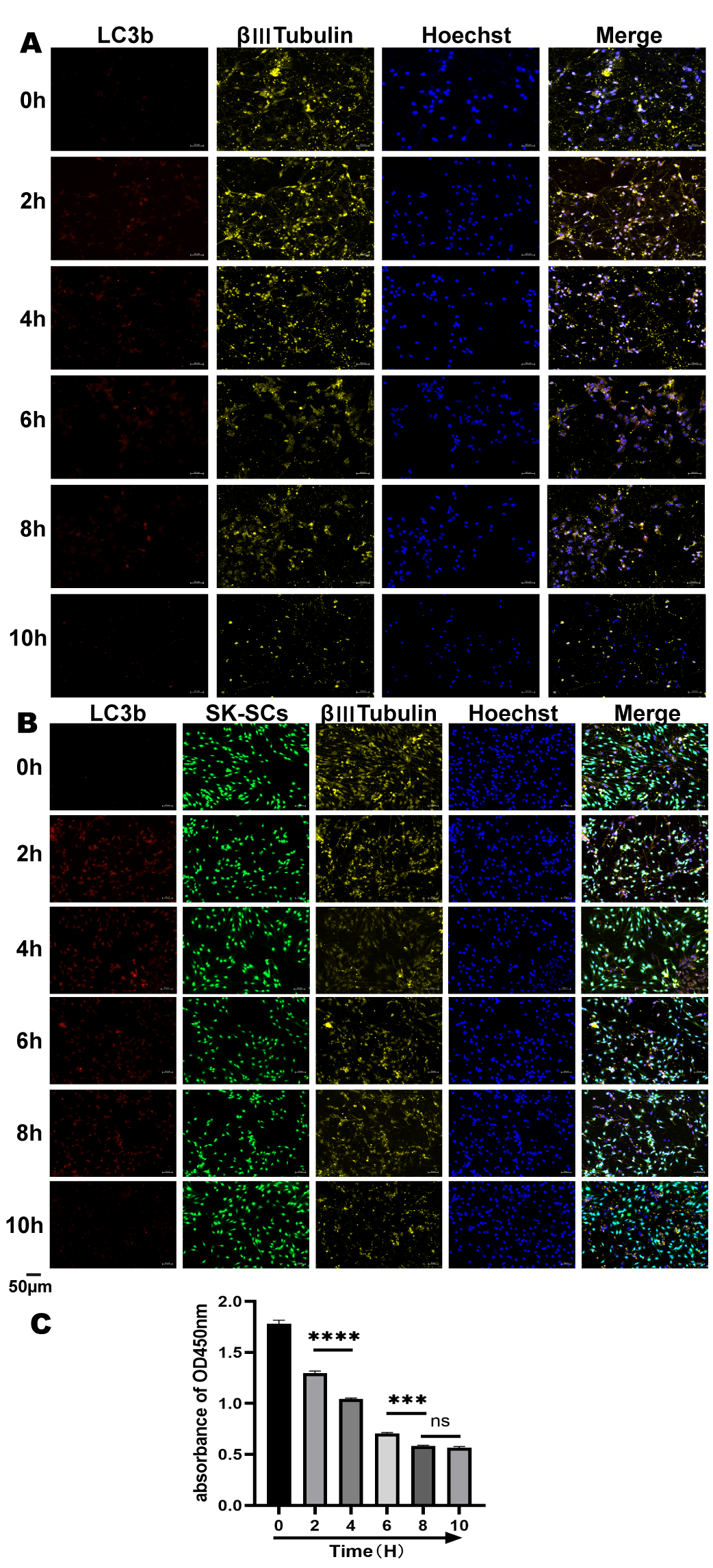

Supplement: Supplementary file 4 — supplementary fig 3 [file 41419_2021_3967_MOESM4_ESM.tif]

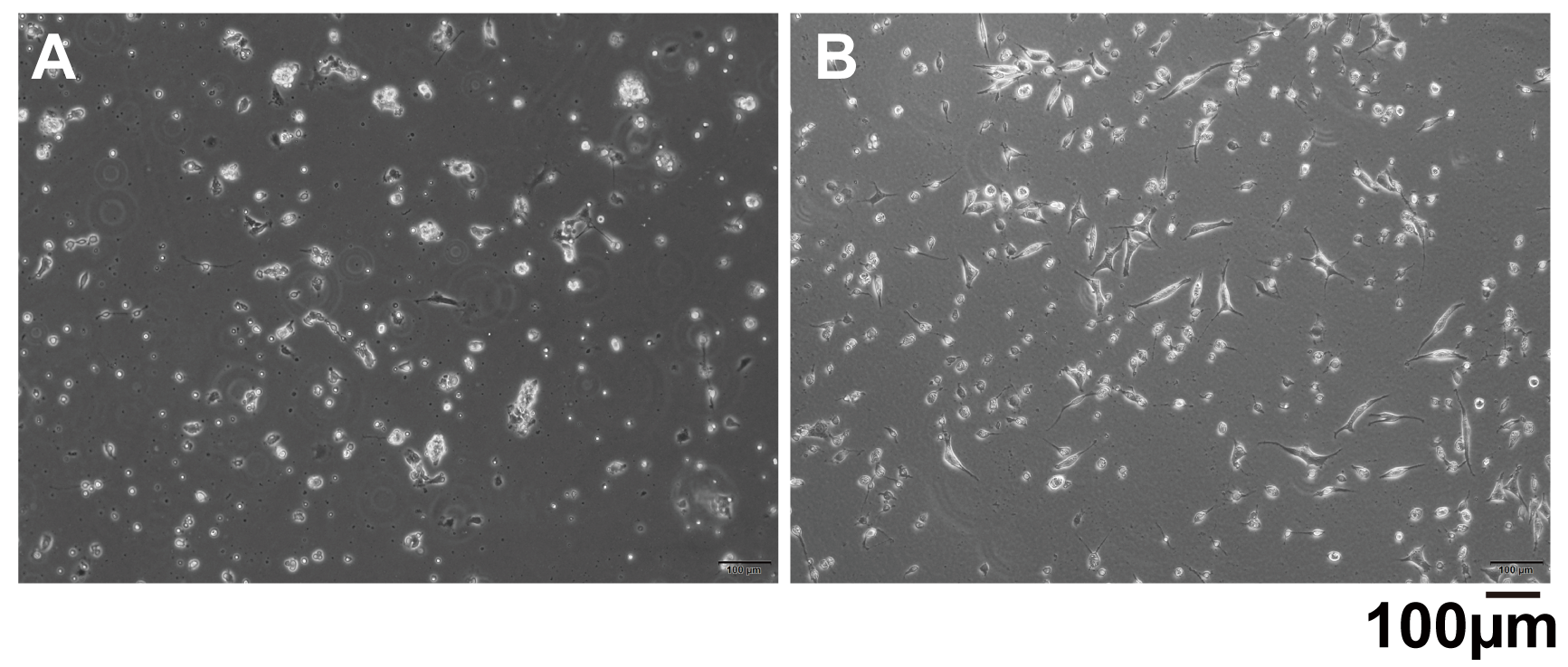

Supplement: Supplementary file 5 — supplementary fig 4 [file 41419_2021_3967_MOESM5_ESM.tif]

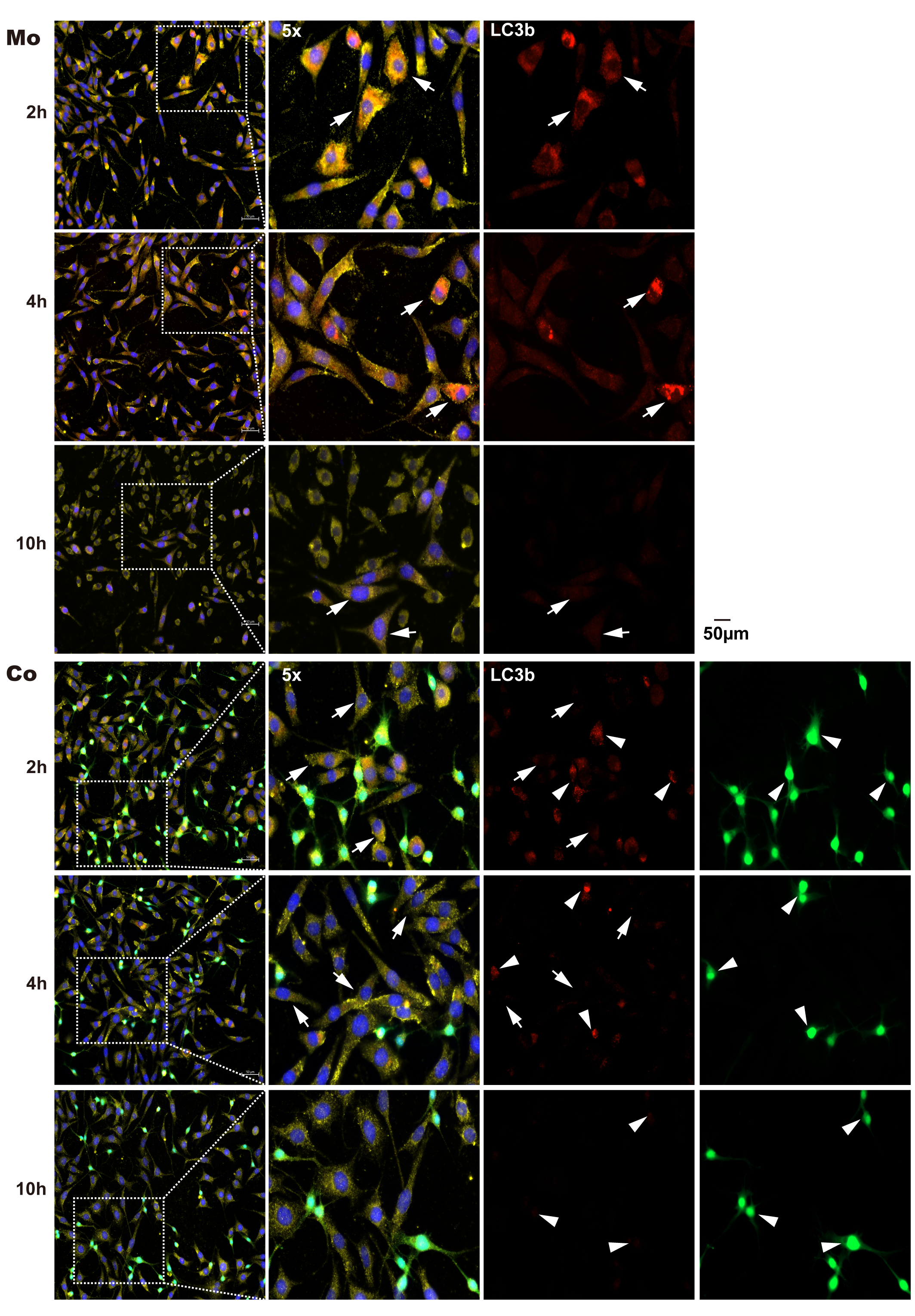

Supplement: Supplementary file 6 — supplementary fig 5 [file 41419_2021_3967_MOESM6_ESM.tif]
