# Supplementary material for: SKP-SCs transplantation alleviates 6-OHDA-induced dopaminergic neuronal injury by modulating autophagy
Source: Cell Death Dis. 2021 Jul 5;12(7):674. doi: 10.1038/s41419-021-03967-3 (PMC8257782; doi:10.1038/s41419-021-03967-3)
Supplement: Supplementary file 7 — Table_Revision [file 41419_2021_3967_MOESM7_ESM.docx]

| **Antibodies** | **Manufacturer** | **Host species** | **Working dilution** | | |
| --- | --- | --- | --- | --- | --- |
|  |  |  | **wb** | **IHC** | **ICC** |
| Tyrosine Hydroxylase | Abcam,ab137869 | Rabbit | 1:1000 | 1:300 | 1:300 |
| Tyrosine Hydroxylase | Abcam,ab76442 | Chichen |  | 1:1000 |  |
| NeuN | Abcam,ab104224 | Mouse |  |  | 1:1000 |
| LC3b | Abcam,ab192890 | Rabbit | 1:1000 | 1:1000 | 1:1000 |
| βⅢ-tubulin | Sigma,T5076 | Rabbit |  |  | 1:1000 |
| Synaptotagmin | Abcam,ab13259 | Mouse |  | 1:200 |  |
| GAPDH | Abcam,ab181602 | Rabbit | 1:10000 |  |  |
| SQSTM1/p62 | Abcam,ab207305 | Rabbit | 1:1000 |  |  |
| Phospho-AMPKα (Thr172) | CST, 8208 | Rabbit | 1:1000 |  |  |
| Phospho-ULK1(Ser555) | CST,97094 | Rabbit | 1:1000 |  |  |
| Phospho-p70S6Kinase | CST,8209 | Rabbit | 1:1000 |  |  |
| Alexa Fluor 568 | Thermo Scientific,11011 | Goat |  | 1:1000 | 1:1000 |
| Alexa Fluor 647 | Thermo Scientific,21235 | Goat |  | 1:1000 | 1:1000 |
| Alexa Fluor 647 | Abcam,ab150175 | Goat |  | 1:1000 |  |
| Secondary Antibody, HRP | Thermo Scientific,21234 | Goat | 1:1000 |  |  |
| **Materials** |  |  |  |  |  |
| DMEM/F12 | Thermo Scientific,11320–033 |  |  |  |  |
| L-glutamine | Thermo Scientific,25030081 |  |  |  |  |
| FBS | GIBCO,16140071 |  |  |  |  |
| Trans-Retinoic Acid | Sigma-Aldrich, PHR1187 |  |  |  |  |
| Accutase | Thermo Scientific, A1110501 |  |  |  |  |
| Poly-L-Lysine | Sigma, P4832 |  |  |  |  |
| Laminin | Sigma, L2020 |  |  |  |  |
| Neurobasal | Thermo Scientific, 21103049 |  |  |  |  |
| B27 Supplement | Stemcell,05711 |  |  |  |  |
| GlutaMAX™ Supplement | Thermo Scientific, 35050061 |  |  |  |  |
| N2 supplement | Thermo Scientific, 17502048 |  |  |  |  |
| Forskolin | R&D Systems, 1099 |  |  |  |  |
| Heregulin-1β | R&D Systems, 396-HB |  |  |  |  |
| Cell Counting Kit-8 | Abcam, ab228554 |  |  |  |  |
| RIPA Lysis Buffer | EpiZyme, PC101 |  |  |  |  |
| protease inhibitor cocktail | MCE, HY-K0010 |  |  |  |  |
| Phosphatase Inhibitor Cocktail I | MCE, HY-K0021 |  |  |  |  |
| BCA Protein Assay Kit | Beyotime, P0012 |  |  |  |  |
| SDS-PAGE Sample Loading Buffer | Beyotime, P0015L |  |  |  |  |
| Omni-PAGE™Hepes-Tris Gels | EpiZyme, LK212 |  |  |  |  |
| Polyvinylidene difluoride membrane | Merck Millipore, P2938 |  |  |  |  |
| Difco™ skim milk | Dickinson and Company, USA |  |  |  |  |
| ECL Western Blotting Substrate | Tanon™,180-5001 |  |  |  |  |
